# Elucidation of molecular and hormonal background of early growth cessation and endodormancy induction in two contrasting *Populus* hybrid cultivars

**DOI:** 10.1186/s12870-021-02828-7

**Published:** 2021-02-24

**Authors:** Ákos Boldizsár, Alexandra Soltész, Karen Tanino, Balázs Kalapos, Zsuzsa Marozsán-Tóth, István Monostori, Petre Dobrev, Radomira Vankova, Gábor Galiba

**Affiliations:** 1 Department of Plant Molecular Biology, Agricultural Institute, Centre for Agricultural Research, ELKH, Martonvásár, H-2462 Hungary; 2 Department of Plant Sciences, College of Agriculture and Bioresources, University of Saskatchewan, Saskatoon, SK S7N 5A8 Canada; 3 Laboratory of Hormonal Regulations in Plants, Institute of Experimental Botany of the Czech Academy of Sciences, Prague, 165 02 Czech Republic; 4 Festetics Doctoral School, Georgikon Campus, Szent István University, Keszthely, H-8360 Hungary

**Keywords:** Endodormancy, Growth cessation, *PtCBFs*, *PtDAM1*, Gene expression, Plant hormone, *Populus*

## Abstract

**Background:**

Over the life cycle of perennial trees, the dormant state enables the avoidance of abiotic stress conditions. The growth cycle can be partitioned into induction, maintenance and release and is controlled by complex interactions between many endogenous and environmental factors. While phytohormones have long been linked with dormancy, there is increasing evidence of regulation by *DAM* and *CBF* genes. To reveal whether the expression kinetics of *CBFs* and their target *PtDAM1* is related to growth cessation and endodormancy induction in *Populus*, two hybrid poplar cultivars were studied which had known differential responses to dormancy inducing conditions.

**Results:**

Growth cessation, dormancy status and expression of six *PtCBFs* and *PtDAM1* were analyzed. The ‘Okanese’ hybrid cultivar ceased growth rapidly, was able to reach endodormancy, and exhibited a significant increase of several *PtCBF* transcripts in the buds on the 10th day. The ‘Walker’ cultivar had delayed growth cessation, was unable to enter endodormancy, and showed much lower *CBF* expression in buds. Expression of *PtDAM1* peaked on the 10th day only in the buds of ‘Okanese’. In addition, *PtDAM1* was not expressed in the leaves of either cultivar while leaf *CBFs* expression pattern was several fold higher in ‘Walker’, peaking at day 1. Leaf phytohormones in both cultivars followed similar profiles during growth cessation but differentiated based on cytokinins which were largely reduced, while the Ox-IAA and iP7G increased in ‘Okanese’ compared to ‘Walker’. Surprisingly, ABA concentration was reduced in leaves of both cultivars. However, the metabolic deactivation product of ABA, phaseic acid, exhibited an early peak on the first day in ‘Okanese’.

**Conclusions:**

Our results indicate that *PtCBFs* and *PtDAM1* have differential kinetics and spatial localization which may be related to early growth cessation and endodormancy induction under the regime of low night temperature and short photoperiod in poplar. Unlike buds, *PtCBFs* and *PtDAM1* expression levels in leaves were not associated with early growth cessation and dormancy induction under these conditions. Our study provides new evidence that the degradation of auxin and cytokinins in leaves may be an important regulatory point in a CBF-DAM induced endodormancy. Further investigation of other *PtDAMs* in bud tissue and a study of both growth-inhibiting and the degradation of growth-promoting phytohormones is warranted.

**Supplementary Information:**

The online version contains supplementary material available at 10.1186/s12870-021-02828-7.

## Key message

Differential timing and expression levels of the key regulatory genes *CBFs* and *DAM1* in buds and down-regulation of cytokinins and IAA, and ABA metabolism in leaves might be involved in the regulation of growth cessation and dormancy development in vegetative buds of contrasting *Populus* cultivars differentially sensitive to low night temperature.

## Background

The synchrony of the plant with its environment enables adapted temperate perennial plants to avoid injury. In these northern, temperate regions, growth cessation is a necessary pre-requisite to cold acclimation and subsequent freezing stress resistance [1, 2]. The growth cycle is regulated by dormancy and in turn, dormancy is governed by both inherent but also environmental factors. While shortening photoperiod has long been known as the most important driver to woody plant dormancy induction [3–6], the temperature has also been and is increasingly recognized as a strong mediator of this response [7, 8] for a review see Tanino et al. (2010) [9]. With global warming, more attention is being paid to temperature and its impact on the dormancy cycle. In this regard, research on forest and agroforest tree species have increasingly highlighted the impact of temperature on dormancy [10–13] and photosynthetic capacity [14]. In North America, with its wide adaptation and fast growth, *Populus* hybrids are the major agroforestry tree of choice in managed lands. Evaluating the impact of future climate change on *Populus* dormancy cycle is important to select cultivars which are better adapted to fluctuating temperatures.

Dormancy in temperate trees is divided into three phases: paradormancy, endodormancy and ecodormancy [15]. Bud dormancy is defined as ‘the temporary suspension of visible growth of any plant structure containing a meristem’ [15]. Paradormancy is defined as growth cessation controlled by physiological factors within the plant but external to the affected structure, endodormancy is defined as growth cessation controlled by physiological factors internal to the affected structure, and ecodormancy represents growth cessation controlled by environmental factors external to the plant [16]. Thus, the various types of dormancy in plants constitute a vast field of study. However, because of the impact of the autumn dormancy induction period on other components of the annual growth cycle [17], and the demonstration that temperature mediates timing and depth of dormancy in *Populus* hybrids [13], in this paper, we will focus on these two aspects.

Furthermore, excellent review papers have focused on the molecular changes, gene regulatory pathways, and hormonal regulations during dormancy [18–25], but relatively less is known about the potential role of C-Repeat Binding Factors (CBFs) in dormancy and Dormancy-Associated MADS-box (DAM) genes in *Populus* hybrid cultivars widely used in agroforestry systems.

*CBF* genes, first described in *Arabidopsis* [26–28], are among the best-characterized plant transcription factors involved in plant abiotic stress tolerance, especially in cold acclimation. The expression of the dehydration-responsive element binding (DREB) protein/C-repeat binding factor gene (*CBF*) is rapidly induced by low temperature. The encoded proteins bind to the CRT/DRE (C-repeat/dehydration responsive element) regulatory DNA motif in the promoters of cold-responsive genes [29], thus inducing their expression, which results in an enhanced cold or frost tolerance [30]. *CBF*s have been described in a huge number of species, both mono- and dicots. Usually several or many gene family members have been identified in one species. Moreover, the number of *CBF* genes may vary even in the same species, in a genotype-dependent manner (copy number variation). Many *CBF*s were described in the monocotyledonous cereals (some 40 in bread wheat /*Triticum aestivum/*, 20 in barley /*Hordeum vulgare/*). Although fewer were described in woody species, their genome also encodes several (3–6) *CBF* genes. Their involvement in cold adaptation has also been confirmed. A recent review [24] summarizes the genetic regulation of cold hardiness in trees.

It is becoming more evident that *CBF* genes are also involved in dormancy regulation, especially in the development of endodormancy [31–34]. Benedict et al. (2006) studied the kinetics and tissue specificity of 4 *CBF*s identified from *Populus balsamifera subsp. trichocarpa* and concluded that *CBF*s are involved in dormancy development and that their differential expression ensures specific roles for these ‘master-switches’ in the different annual and perennial tissues [35].

The existence of CBF – DAM – dormancy ‘pathway’ has been suggested and, at least partially, shown by several studies in Japanese pear [36, 37] and Japanese apricot [38]. In his review, Horvath (2009) proposed a theoretical model, ‘which can be developed that could serve as a paradigm for further testing’ [39]. Wisniewski et al. (2011) demonstrated that transgenic apple (*Malus* x *domestica*) plants, expressing a peach (*Prunus persica*) *PpCBF1* gene showed not only an increased level of freezing tolerance, but also a modified response to short photoperiod, leading to the early onset of dormancy, early leaf senescence, and delayed bud break [31]. As a next step Wisniewski et al. (2015) analyzed *CBFs*, *DAMs*, *RGLs*, and *EBB* transcription factor genes, involved in the regulation of dormancy [32]. The expression of several apple *DAM* genes - already associated with dormancy development in woody *Rosaceae* plants - exhibited different expression patterns. CBF binding sites identified in the apple *DAM* promoters led to the suggestion of a regulatory model connecting *CBFs* and *DAM*s expression to endodormancy development [32]. *DAM* genes were first identified in *Prunus.* A mutant peach, called ‘ever-growing’, was unable to enter endodormancy even when plants were exposed to short photoperiods or low temperatures [40–42].

*DAM* genes are members of the type II (MIKCc) subfamily of MADS-box transcription factors. Their sequences contain four major domains, the MADS-box (M), intervening (I-), keratin-like (K-), and C-terminal (C-) domain. These domains are responsible for DNA binding, protein dimerization, complex formations, and transcriptional regulation. A detailed structural and functional characterization of *DAM* genes can be found in the reviews published by Horvath (2015) and Falavigna et al. (2019) [43, 44]. In this latter publication, a model is proposed, introducing the molecular network of the regulatory genes involved in the dormancy cycle.

Expression patterns of the *DAM* genes were related to endodormancy and were mainly presented in the *Prunus* genus, among them peach (*P. persica*) ever-growing [45] and peach cultivars [46], Japanese apricot (*P. mume*) [47], and also in apple [48] or Japanese pear (*Pyrus communis*) [49, 50]. *DAM* gene expression appears to be linked to the stage of dormancy (see Falavigna et al. (2019) for a review [44]). In most species, *DAM* gene expression is induced during the dormancy induction period but may also be involved in maintenance and release [46]. Based on amino acid sequence, poplar *DAM1* and *DAM2* expression were most closely associated with leafy spurge *MADS 27–29* and unlike the other *DAM* genes, *DAM1* and *DAM2* were upregulated by dormancy inducing short-day conditions in poplar (Chen (2008) [51] as cited by Horvath et al. (2010) [52]). Interestingly in a later transcriptome study these results were not confirmed. Howe et al. (2015) examined several *DAM*-like genes that were downregulated during endodormancy [53]. One of the examined genes was the Potri.002G105600, but in this study, the authors did sampling only once per month.

Analysis of transgenic plants showed *CBF* genes were also involved in the regulation of endodormancy. The ectopic expression of a peach *PpCBF1* gene in apple resulted in short-day induced dormancy and increased cold hardiness [31], and affected the expression levels of apple *MdDAM1* and *MdDAM3* genes in buds [32]. Li et al. (2019) analyzed pear (*Pyrus pyrifolia*) *CBF* and *DAM* genes and found multiple *CBF* genes selectively regulate *DAM* genes and participate in endodormancy regulation [37]. Interestingly, this group found that “PpCBF1-PpDAM2 regulon mainly responds to low temperature during endodormancy regulation, with further post-translational regulation by PpICE3”. In addition, the expression of *ParCBF1* was found to be in close association with the decreasing ambient temperatures in apricot (*Prunus armeniaca*), and the expression levels of *ParDAM5* and *ParDAM6* changed according to *ParCBF1* expression rates [54].

Molecular evidence also supports the CBF - DAM connection. The presence of CBF transcription binding sites was reported in the putative promoter regions of the leafy spurge *DAM* genes [52]. A model, illustrating a potential interaction between DREBs (CBFs) and *DAMs* was subsequently suggested [55]. An interaction of PpCBF2 protein with the promoter of *PpMADS13–1* gene was shown in pear by transient reporter assay [56]. Also in pear, yeast one-hybrid and transient assays showed that PpCBF2 enhanced *PpDAM1* and *PpDAM3* transcriptional activity during the induction of dormancy [38]. Zhao et al. (2018) showed that *P. mume* CBFs can bind to the *PmDAM6* promoter via alternative binding sites and activate its expression [33, 34]. Japanese pear *PpCBF*s were able to induce the expression of *PpDAM1–1* and *PpMADS13–3* genes in transient reporter assays [56]. Different biochemical methods revealed that pear *PpCBF2* and *PpCBF4* genes are able to bind to the promoter of *PpDAM1* gene, activating its expression, and revealed that *PpCBF1, PpCBF2, PpCBF3, PpCBF4* genes can activate *PpDAM3* gene [37]. These results demonstrate the CBF - DAM signalling pathway is involved in endodormancy development and also demonstrate a certain level of *CBF* functional redundancy.

Herein we use a system of two contrasting *Populus* hybrid cultivars differing in growth cessation and dormancy acquisition which were previously distinguished based on low night temperature under short photoperiod. The hypothesis that growth cessation and dormancy induction is linked to leaf phytohormone levels, bud *PtDAM1* and bud *PtCBFs* gene expression in *Populus* will be evaluated.

## Results and discussion

In our previous work [13] we studied the impact of temperature on growth cessation, dormancy development, and cold acclimation of four poplar cultivars. These temperature regimes changed the kinetics of dormancy development patterns with the 18/3 °C treatment inducing the widest separation of dormancy depth. Therefore, to elucidate if there is a relationship between the expression levels of *CBF* genes and dormancy in poplar, two cultivars differing in their dormancy acquirement based on night temperature responses were tested under short-day conditions in our current research.

### Dormancy development

#### Growth cessation

**Fig. 1:**
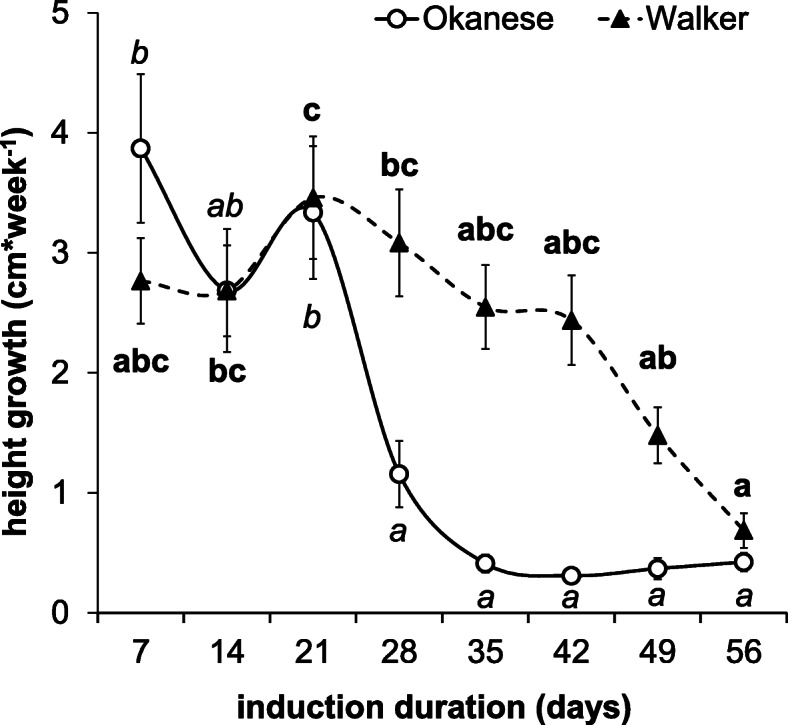
Shoot length increments (cm*week^1^) in the Okanese (circle) and Walker (triangle) cultivars under SD conditions (12/12 h, light/dark period) at 18/3 °C temperature during the first 30 days, then 10/14 h for an additional 30 days. Error bars represent the ±SEM. *N* = 27. The values indicated by different letters are significantly different at *P* < 0.05 level from each other

Growth cessation is the first indication of dormancy induction [57] and was induced in both genotypes (Fig. 1). Consistent with Kalcsits et al. (2009), a significantly earlier and steeper drop could be observed in the ‘Okanese’ compared to ‘Walker’ cultivar, between the 3rd and the 4th week of dormancy inducing conditions. After the 5th week, no further growth was recorded. ‘Walker’ showed higher growth rate at every time-point, with the exception of the 1st week. By the end of the experiment, no significant difference was found between these two cultivars (0.36 and 0.51 cm * week^− 1^ in Okanese and Walker, respectively).

#### Dormancy induction

**Fig. 2:**
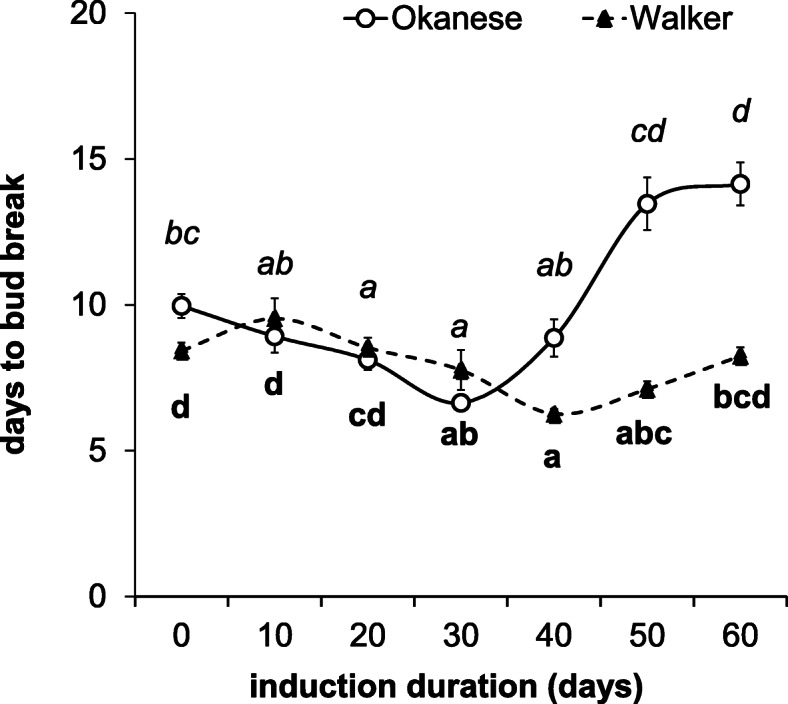
Dormancy development (characterized as days to bud break) of Okanese (circle) and Walker (triangle) cultivars under SD conditions (12/12 h, light/dark period) at 18/3 °C temperature during the first 30 days, then 10/14 h for an additional 30 days. Error bars represent the ±SEM. *N* = 40. Different letters indicate statistically different (P < 0.05) number of days for the given cultivar

While growth cessation was a more sensitive indicator, significant differences in the number of days to bud break were found between the two cultivars from day 40 (Fig. 2). Okanese buds took 10 days to break bud at Day 0, while at the end of the experiment (i.e. on the 50th and 60th day) this value was increased and levelled off at 13.5 and 14.1 days (respectively). Conversely, the duration of bud break was hardly changed in ‘Walker’ over the whole 60 days treatment period, just a slight fluctuation was recorded (Fig. 2). No difference was detected between the first and the last days (days to bud break: 8.4 and 8.2 days, respectively).

The depth of dormancy was reflected by the parameter ΔDBB (Differences between the first and last Days to Bud Break) and dormancy was not induced at all in ‘Walker’ (ΔDBB = 0.2). Conversely in ‘Okanese’, the dormant state started to be induced after 30 days. At the end of the experiment, Okanese had a ΔDBB of 4.1 (Fig. 2). The data on growth cessation rate and the bud break analysis indicates that ‘Okanese’ reached a deeper dormant state than ‘Walker’. Our results are consistent with the outcome of Kalcsits et al. (2009) [13], who reported characteristic differences between these two cultivars – ‘Okanese’ was shown to be more capable of endodormancy development under the 18/3 °C day/night temperature treatment under 12 h and 10 h daylengths although a larger difference was found between the two cultivars (ΔDBB: 13.9) in that study.

### Expression pattern of *CBF* genes

The expression patterns of six *CBF* genes were recorded over the whole experiment. Samples were collected from leaf and bud tissues every ten days, taking into account the circadian rhythms of many *CBF*s, in the same period of the day, i.e. 4–6 h after the start of the light period. The expression of each gene in a given time-point was normalized to the level measured at the beginning (i.e. on the 0 day) of the given treatment.

**Fig. 3:**
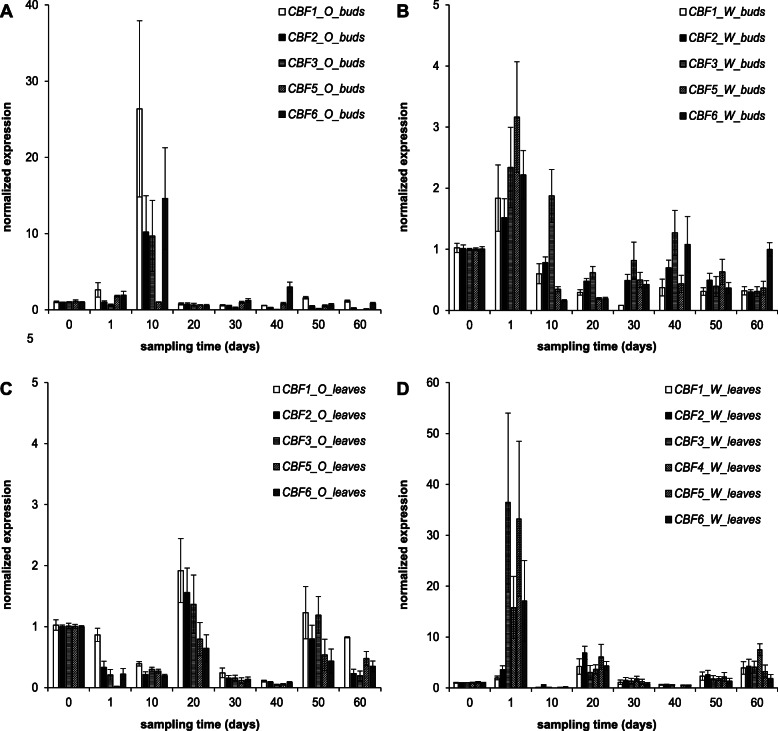
Relative expression of *PtCBF1, PtCBF2, PtCBF3, PtCBF4, PtCBF5* and *PtCBF6* genes in the Okanese and Walker cultivars in buds (panel **a**-**b**) and in leaves (panel **c**-**d**). Mean expression values were normalized per the expression level at the zero sampling time-point, separately for each genotype. Error bars represent the ±SEM originating from 3 biological and 3 technical replicates

Differences in the kinetics and spatial localization of the overall *CBF* expression were observed between the two cultivars. The highest levels of *CBF* expression across the entire experiment were recorded in the bud tissue, isolated from ‘Okanese’ on the 10th day (Fig. 3a) and on the first day in ‘Walker’ leaf samples (Fig. 3d). The expression levels in ‘Okanese’ poplar buds peaked at the 10th day and were at least an order of magnitude (10–20 fold) higher than in ‘Walker’ buds, and at any other time during the experiment for all the *CBF*s (with the exception of *PtCBF*5). There was differential expression of bud *PtCBFs* between the two cultivars in that *PtCBF1* and *PtCBF5* showed the highest and lowest expression in ‘Okanese’ buds, respectively, while the reverse was observed in ‘Walker’. In ‘Walker’ leaves on the 1st day, *PtCBF1* and *PtCBF2* expression levels were roughly equivalent to ‘Okanese’, however, ‘Walker’ leaf expression of *PtCBF3, PtCBF4, PtCBF5 and PtCBF6* spiked on the 1st day and were 150–200 times greater than ‘Okanese’ (Fig. 3c, d).

The expression patterns of the unique *CBF* genes are described in detail in the Supplemented Fig. 1. In bud tissue, *PtCBF2*, *PtCBF3* and *PtCBF5* were induced only in the beginning of the experiment, on the 10th day, while *PtCBF1* and *PtCBF6* were induced not only at the beginning but also at the end of the treatment, on the 50th and 60th (*PtCBF1*) or on the 40th day (*PtCBF6*). The induction level was always an order of magnitude higher in the ‘Okanese’ buds compared to ‘Walker’ for each *CBF*. The repression of *CBF* genes was more pronounced in the ‘Walker’ buds. A repressed period was recorded in the middle of the experiment for *PtCBF1*, *PtCBF5* and *PtCBF6* genes in ‘Walker’ buds, while only one repressed stage was found in ‘Okanese’ in the mid period of *PtCBF*3 expression (Supp. Figure 1A, D, E and C).

By contrast in leaf tissue, two induction waves could be observed in the leaf samples for all *CBF*s in ‘Walker’: the first was at the beginning (on the 1st and 20th day), while the second was at the end (50th and 60th day). Induction waves were also found in ‘Okanese’ leaves, but in the opposite direction, since repression of all *CBF*s was detected in the period 1st-10th and 30th–40th and finally on the 60th day. It is interesting to note the differential responses between the cultivars in leaf tissues in that CBF induction was found in ‘Walker’ leaves, while repression was found almost in every case in ‘Okanese’ leaves (Supp. Figure 1F-K). Thus, these two cultivars had similar but opposite *PtCBF* expression under dormancy inducing conditions based on buds or leaves.

Differences in the *CBF* expression kinetics and levels measured in the meristematic (bud, stem) and leaf tissues were studied in several cases in woody plants, among them poplar. Benedict et al. (2006) described different *CBF* induction patterns in *P. balsamifera* Ss*p. trichocarpa* showing that all four *PtCBF*s are cold-inducible in leaves, while only two (*PtCBF1* and *PtCBF3)* were cold induced in the stem [35]. Under a short-expression period (24 h), they concluded, ‘the perennial driven evolution of winter dormancy led to the development of specific roles for abiotic stress response regulators, such as the *CBF*s, in annual and perennial tissues’. *CBF* expression was followed in leaf and leaf bud tissues in *Prunus mume* during one year by Zhao et al. (2018) [33]. They also found a differential gene expression pattern for all six *CBF*s studied, with specific induction kinetics. In that study, all six *CBFs* were induced in vegetative buds, in the cold period (November – January); *PmCBF4*, *PmCBF5* and *PmCBF6* being the most intensively expressed. These three *CBF*s were also the most induced in the leaf tissues. But interestingly, in leaves, the highest expression for all 6 *CBF*s was recorded during the warmest period, from June to July. This finding is in accordance with our results, i.e. that the *CBF* expression was much more intense in leaves of the non-dormant cultivar, may indicate that their role in the development in dormancy is organ-specific. Six *PmCBF*s in 7 different organs were determined in *P. mume* [33]. The induction levels were high in stems, moderate in flower buds, leaf buds, and leaves, poor in flowers, fruits, and seeds.

Gene duplication and multiplication produced a large number of *CBF*s in many species. This redundancy makes possible the divergence of functionality, and the possibility for fine-tuning of adequate response for any environmental stimuli, such as stress. As mentioned above, 6 *CBF*s encoded in the *P. mume* genome exhibited different expression kinetics during the year: *PmCBF1, PmCBF2,* and *PmCBF3* were up-regulated in the stem tissues not only in the cold period but also in late spring [34]. Additionally, low temperature up-regulated 8 *CBF*s in *Prunus mume* which subsequently induced all six *DAM* genes resulting in dormancy development [36]. Under natural dormancy induction conditions, 3 out of 4 *CBF*s showed similar expression trends in *Pyrus pyrifolia* bud tissues, while *PpCBF1* showed a different induction kinetic [37]. During an artificial chilling test, *PpCBF1* was the only *CBF* highly expressed, while *PpCBF2* was repressed intensively, and the levels of *PpCBF3* and *PpCBF4* were undetectable.

These results show that although *CBF* expression kinetics may be similar, differences in the individual expression patterns can be distinguished. Shortening the light period by 2 h/day to account for the variance in nature (at the same temperature regime) may have caused a moderate functional polymorphism in our experiment. *PtCBF4* was detectable only in ‘Walker’ leaves, while *PtCBF1* and *PtCBF6* were the most intensively expressed genes in ‘Okanese’ buds. Whether they have different functions, as was suggested for *PpCBF4* [37] in pear, is still unclear. It is also remarkable that *PtCBF5* was the only gene which was not induced during the CBF-burst on the 10th day in ‘Okanese’ buds but was the most intensively up-regulated in ‘Walker’ buds on the 1st day. Therefore, we assume *PtCBF5* is not related to dormancy development.

Leaf samples of *Populus balsamifera ssp. trichocarpa* genotypes originating from northern and southern populations were examined [58]. A growth chamber study showed all *PtCBF* genes were induced by cold, indicating functional redundancy. On the other hand, under field conditions, a more diverse gene expression pattern was described. The expression of *PtCBF*s increased as the growing season progressed, but among the six genes, only *PtCBF3* was marginally differentially expressed across latitudes. In our experiment, leaf samples also showed a certain level of functional polymorphism, but the most common outcome of the two systems is that in leaves, no dormancy dependent expression pattern was found, such a relation was present only in the bud tissues.

### *PtDAM1* identification and its expression kinetics

**Fig. 4:**
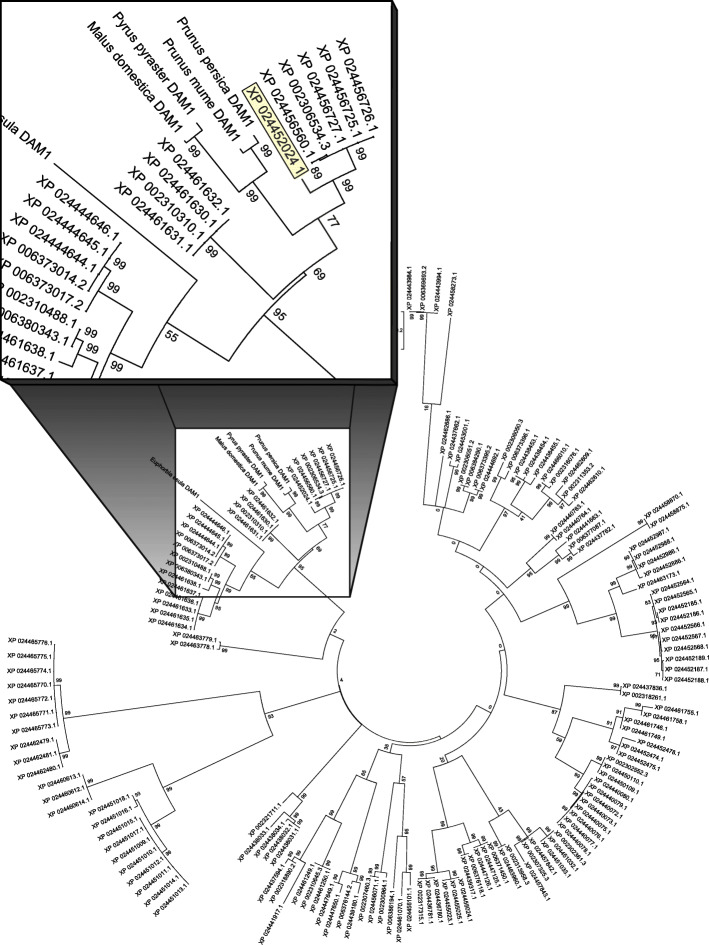
Phylogenetic relationships of PtDAM1 and other DAM proteins containing K-box and SRF-TF domains identified in woody plants. Magnified box shows DAM1 proteins from different plant species where the putative PtDAM1 *Populus* sequence (XP 024452024.1) is highlighted

**Fig. 5:**
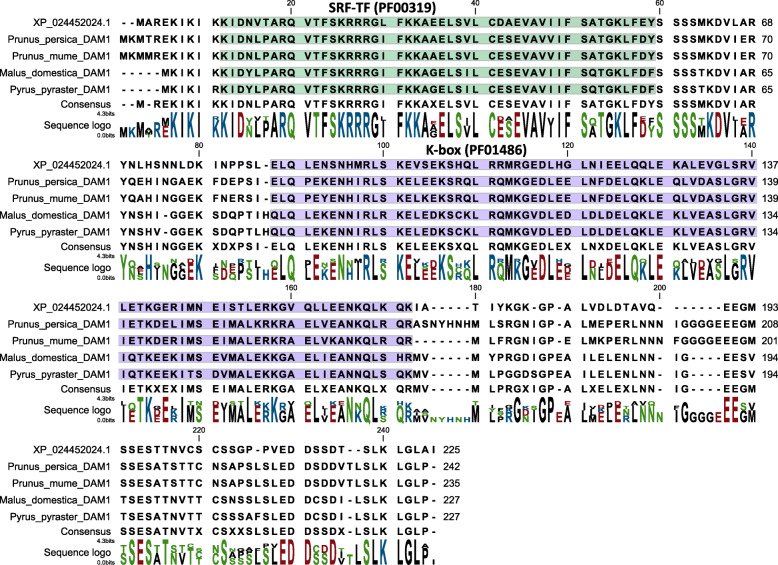
Multiple alignment of DAM1 protein sequences. 4 representative DAM1 protein sequences of various tree species and the candidate PtDAM1 (XP_024452024.1) were aligned by the MUSCLE alignment method. The consensus is indicated below the sequences and the homology visualized by sequence logo. The characteristic SRF-TF (PF00319) and K-Box (PF01486) motifs are highlighted on the alignment with green and purple, respectively

*DAM*s (*Dormancy-Associated MADS-Box*) are well-characterized genes in perennial plants, associated with various components of the dormancy cycle but particularly dormancy induction. DAM sequences had already been published in woody plants, all containing K-box and SRF-TF motifs [33, 59, 60]. The *P. trichocarpa* genome has been sequenced [61], however, it is still poorly annotated. We have found 151 candidates for the *DAM* genes. From these, we suggested the XP_024452024.1 protein entry (available at the NCBI protein database: https://www.ncbi.nlm.nih.gov/protein/) as a putative *PtDAM1* product (Figs. 4 and 5). The XP_024452024.1 entry is corresponds to older versions as MADS7, Potri.002G105600 at the PopGenIE database: https://popgenie.org/). Howe et al. (2015) studied transcriptome changes during endodormancy induction by microarray in *P. trichocarpa* and found several *DAM-like SVP* genes were differentially expressed but were downregulated during endodormancy [53]. Since sampling was conducted on a once per month basis, it is not clear if upregulated peaks were missed.

**Fig. 6:**
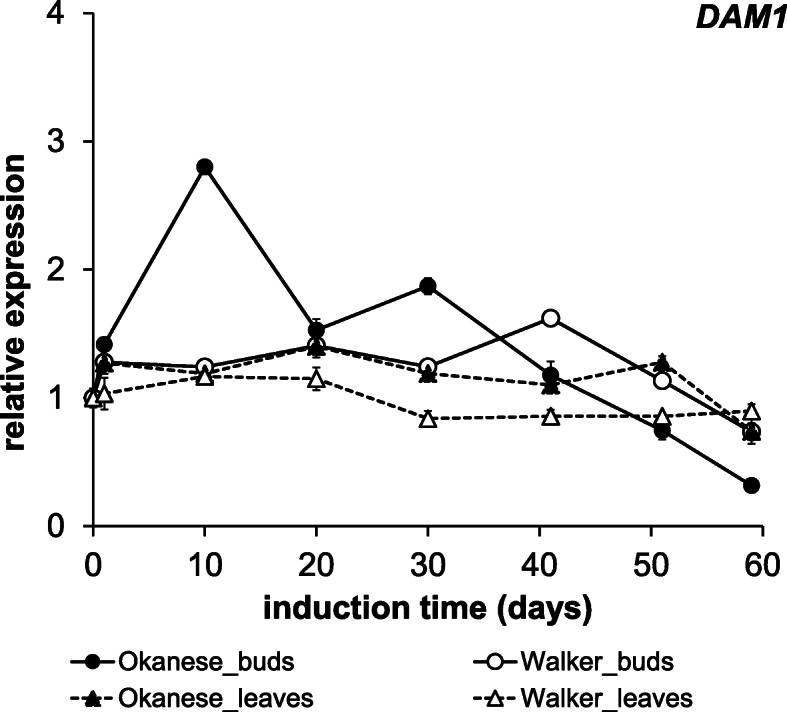
Relative expression pattern of *PtDAM1* gene in the buds and leaves (circle and triangle, respectively) of the Okanese (solid) and Walker (open) poplar cultivars. Error bars represent the SD of the technical replicates

Having identified a *PtDAM1* gene in *Populus*, we decided to evaluate its potential role in dormancy development, using cultivars known to be differentially responsive to night temperatures. Therefore, primers were developed to study the encoding *PtDAM1* gene expression. Compared to the first sampling day, mild up-regulation of the identified putative XP_024452024.1 sequence was recorded in ‘Okanese’ leaves through the experiment, while lower induction was found in ‘Walker’. *PtDAM1* was repressed from the middle of the experiment (Fig. 6) and the expression of *PtDAM1* was almost unchanged throughout the 60 days in leaf tissues. The bud tissues showed much more pronounced induction than the leaf tissues. In more dormant ‘Okanese’, the maximum expression (2.8-fold) was recorded on the 10th day then the induction gradually declined. Repression was recorded in both cultivars at the end of the treatment. *PtDAM1* induction in buds was weaker in the first half of the treatment in ‘Walker’ (1.1–1.6-fold induction) which did not enter endodormancy.

Similar expression trend for *PmDAM1* gene was described in Japanese apricot (*Prunus mume*) bud tissue, but differently in the leaf samples [47]. In the vegetative buds, expression of *PmDAM1* (as well as *PmDAM2* and *PmDAM3*) was upregulated from June to July, i.e. long before the start of growth cessation, then expression started to decrease. We also showed an initial *PtDAM1* induction in our system, well before the start of growth cessation, or dormancy development. We found no characteristic changes in leaf tissues, however, in *Prunus mume,* different kinetic patterns were described in this organ [47]. Two seasonal expression trends were shown for *P. mume DAMs*, *PmDAM1* (together with *PmDAM2* and *PmDAM3*) was rapidly up-regulated in spring, being gradually down-regulated in autumn. This difference in the expression in leaf tissue might be explained by the two different experimental systems. In other studies in peach (*Prunus persica*), differential *DAM* gene expression appeared to be related to dormancy induction or fulfillment of the chilling requirement phases. Based on the ever-growing peach mutant system, Li et al. (2009) reported *DAM1*, *DAM2* and *DAM4* were the most likely candidates associated with growth cessation and dormancy induction [45]. Using the same system, Yamane et al. (2011) showed under both field and controlled environment conditions and in leaves and stems, *DAM5* and *DAM6* gene expression levels were up-regulated during endodormancy induction and downregulated during endodormancy release which appeared to be tied to chilling requirement satisfaction [62]. Furthermore, *DAM5* and *DAM6* gene expression levels were higher in high chill cultivars and reduced with chilling requirement satisfaction [63]. *DAM5* and *DAM6* genes were negative regulators of bud break.

### Dynamics of hormone changes during dormancy development

Phytohormones have been long known to be involved in the dormancy cycle [23, 25, 64–72]. Recently, mechanistic relationships between phytohormones and dormancy are being revealed [36, 73, 74].

**Fig. 7:**
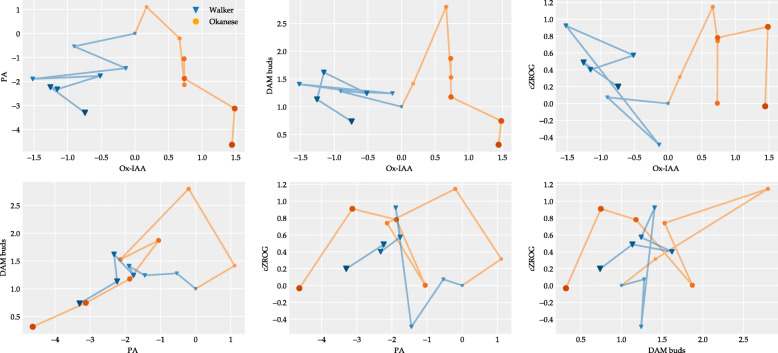
Top four contributing components of the PCA analysis are Ox-IAA, Phaseic Acid, *DAM1*, cis-zeatin riboside-O-glucoside (cZROG), accounting for 26.6, 23.3, 12.3 and 9.9% of the variation, respectively. Each component is plotted in the function of the other three components after standardization. The color saturation with the markers size indicates the sampling time of the poplar cultivars, between 0 and 60 days. Filled triangles and circles show the results obtained for Walker and Okanese cultivars, respectively

**Fig. 8:**
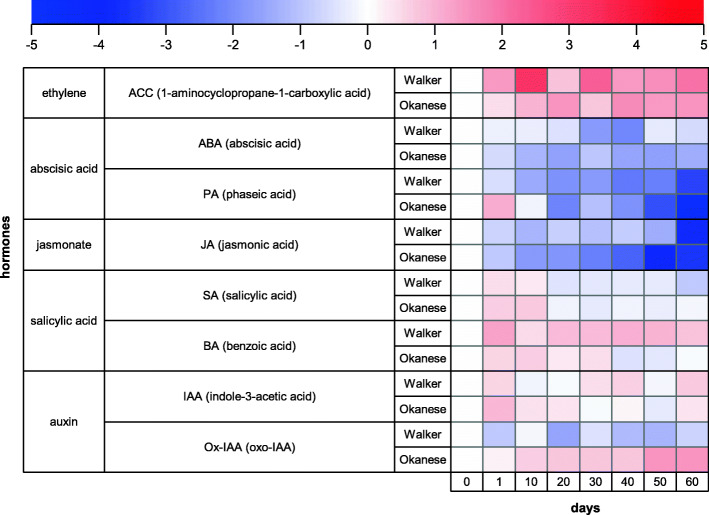
Heatmap of the levels of ethylene, abscisic acid, jasmonate, salicylic acid, auxin and their metabolites in the leaves of Walker and Okanese poplar cultivars. Colour scale represents the log_2_ transformation of the ratio between concentration measured at the given sampling time and concentration measured on day 0

In our study, due to the very small size of poplar buds and only limited capacity of growth chambers, hormone analysis was conducted only in leaf samples. Overall, phytohormonal response in ‘Okanese’ was different than in the ‘Walker’ poplar hybrid cultivar with most significant distinction for Ox-IAA, phaseic Acid, DAM1, *cis*-zeatin riboside-O-glucoside (cZROG) (Figs. 7 and 8). Exposure of poplar plants to short photoperiod and low night temperatures was associated with down-regulation of ABA content in leaves of both genotypes (Fig. 8). However, an early (on the 1st day) transient elevation of the ABA metabolite, phaseic acid, indicated enhanced ABA degradation in the ‘Okanese’ cultivar, suggesting a preceding short-term up-regulation of ABA content early after temperature drop. This assumption is supported by the report on transient up-regulation of ABA in cold-stressed wheat leaves [75]. The ethylene precursor ACC was elevated in both clones. Ruttink et al. (2007) showed ethylene rise preceded ABA during dormancy induction [64]. Jasmonate has been known to be involved in several stress responses [76]. Inactivation of the repressors of JA signaling pathway - jasmonate ZIM-domain (JAZ) proteins, which physically interact with ICE1 and ICE2 transcription factors, results in up-regulation of *CBFs* [77]. *CBF* genes promote gibberellin deactivation and thus growth inhibition [78]. In our study in leaf tissue, JA levels were suppressed in both genotypes during the entire experimental period, and more in ‘Okanese’. However, JA level in leaves need not correlate with its content in buds. Moreover, JAZ inactivation may be achieved by their interaction with DELLA proteins [79, 80], which accumulate at low temperature and are stabilized by gibberellin down-regulation. In contrast to JA, SA levels were increased at the beginning of the experiment, one week longer in ‘Okanese’. This agrees with the positive effect of SA on plant cold tolerance [81]. After the 3rd week, the SA content was unchanged in both cultivars, however, the concentration was lower in the less cold-hardy ‘Walker’. Benzoic acid, the precursor of SA and other phenolic compounds, was elevated during the experiment; in ‘Okanese’ until dormancy initiation, in ‘Walker’ during the whole experiment. These changes demonstrate differences in hormonal dynamics between the clones during leaf senescence (Fig. 8).

The auxin, indole-3-acetic acid (IAA), had varying levels across the 60-day treatment in both cultivars. However, the main IAA catabolite, Ox-IAA, had a more consistent response, being up-regulated in ‘Okanese’ and down-regulated in ‘Walker’, which indicates stronger IAA deactivation in ‘Okanese’ leaves. Dormancy initiation, associated with substantial suppression of growth rate, was accompanied by IAA down-regulation, which was not observed in the non-dormant clone (Fig. 8). Baldwin et al. (2000) showed that while the auxin naphthaleneacetic acid was not required for bud scale development, its absence was critical [82].

**Fig. 9:**
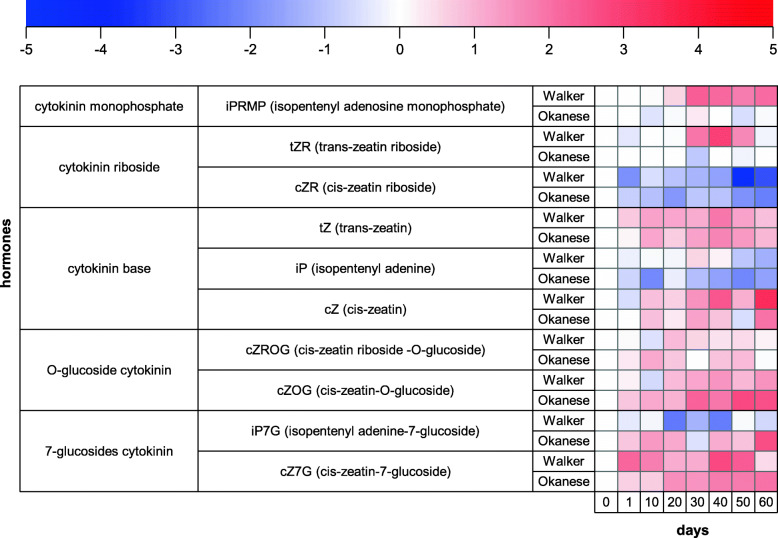
Heatmap of the levels of cytokinin metabolites in the leaves of Walker and Okanese poplar genotypes. Colour scale represents the log_2_ transformation of the ratio between concentration measured at the given sampling time and concentration measured on day 0. The up to down order of the presented hormones is following the metabolic pathway from precursors (iPRMP, tZR, cZR) through active (iP, tZ, cZ) to deactivated (CK O-glucosides and N-glucosides) forms

The whole cytokinin pathway was downregulated in Okanese compared to Walker: the precursors iPRMP and tZR increased only in Walker, the active form (iP) decreased only in Okanese, and the deactivated form iP7G was accumulating in Okanese and decreasing in Walker. Other compounds did not show any major changes between both trees (Fig. 9).

Cytokinin analysis clearly showed that promotion of dormancy in ‘Okanese’ was associated with a general decrease of cytokinin biosynthesis and profound elevation of their deactivation products in leaves (Figs. 7 and 9, Supp. Figure 2). Collectively, these results provide new evidence that the degradation of growth-promoting phytohormones such as IAA and cytokinins may be an important mechanism of endodormancy induction.

### The relation between *PtCBFs* and *PtDAM1* expression, hormone level and the development of dormancy

A *CBF*-burst occurred on the 10th day of the short photoperiod and low night temperature treatment in ‘Okanese’ bud tissues, while in ‘Walker’ *CBF* levels were an order of magnitude lower (Fig. 3). In ‘Okanese’ which was able to enter endodormancy (Fig. 2), *CBF1* had the highest relative expression at the initiation of dormancy. *PtDAM1* expression peaked in ‘Okanese’ exactly on the same 10th sampling day (Fig. 6). By contrast, ‘Walker’ which did not attain endodormancy (Fig. 2) had a lower *CBF* expression on the 1st day (Fig. 3), while *PtDAM1* expression was also low and unchanged during the experiment (Fig. 6). Growth rate started to decline in both cultivars by the 3rd week, but at a much faster rate in ‘Okanese’ (Fig. 1). These findings support the possible relationship between *PtCBF1*, *PtDAM1* induction and endodormancy development.

The dormancy-associated phytohormone, ABA, was surprisingly down-regulated in leaves of ‘Walker’ and even more downregulated in ‘Okanese’. However, the concentration of the ABA degradation intermediate, phaseic acid, increased in ‘Okanese’ while it was reduced in ‘Walker’ and therefore, an ABA induction peak in ‘Okanese’ leaves may have been missed (Fig. 8). Recent evidence indicates a role of DAM1 in activating *NCED3* through binding to its promoter and upregulating ABA biosynthesis in Japanese pear [83]. The same study found high concentrations of ABA can also reduce DAM1 in a feedback regulatory loop. DAM proteins are similar to SVP (Short Vegetative Phase), one of the flowering time regulators in Arabidopsis. In kiwifruit, Wu et al. (2017) [84] performed a transcriptomic analysis and found AcSVP2 may mimic ABA action [85]. They further indicated that SVP2 was mediated by ABA to decrease meristem activity and prevent premature bud break. DAMs also appear to play a regulatory role in the ABA signaling pathway [85]. Thus, there is increasing evidence that *CBF* and *DAM* gene actions are linked with phytohormonal concentration and action in dormancy. The reverse has also been demonstrated in that Knight et al. (2004) earlier showed ABA to upregulate *CBF* expression [86]. Singh et al. (2019) reported that SVL is the ortholog of SVP in aspen (*Populus tremula x tremuloides*), which mediates photoperiodic dormancy induction via callose synthase, operating downstream of ABA [74]. Singh et al. (2018) also showed ABA induced the expression of the *DAM/SVL* gene in hybrid aspen [87]. For an excellent recent review, see Liu and Sherif (2019) [25].

In a recent study, analysis of a transformant hybrid aspen (*Populus tremula* x *tremuloides*) showed that expression of *SVL*, a negative regulator of bud break, was down-regulated in hybrid aspen buds after low temperature treatment. It was noted that nonetheless, *SVL* is similar to *DAM* genes, clustering closer to *SVP* in *Arabidopsis* and apple than to hybrid aspen or peach *DAM* genes [74, 87]. Interestingly, *SVL* induced the expression of callose synthase and negatively regulated the gibberellin pathway. Moreover, CBF14 and CBF15 upregulated the *GA2ox5* gene which deactivates gibberellins in barley [88].

Dormancy is known to be induced primarily by temperature in some fruit species, such as apple and pear [89]. Increasing evidence highlights the role of temperature, especially in the case of northern woody cultivars. While the main regulator of growth cessation and dormancy induction in woody species is short photoperiod, it may be moderated by, and interact with temperature [17]. The increasing confirmation of direct regulation by cold-induced *CBFs* on *DAM* gene expression [34, 37, 56], Niu et al. (2016) provided evidence and proposed a model in which CBF induces *DAM* and DAM downregulates *FT* which then suppresses growth and stimulates the development of dormancy [38]. Liu and Sherif (2019) further outlined a model integrating multiple phytohormonal networks regulated by DAM [25]. Key among them was the direct suppression by DAM of cytokinins, gibberellins and direct activation by DAM of ABA and callose deposition. Our study provides additional evidence that cytokinin and IAA degradation may be an important regulatory mechanism to endodormancy induction.

## Conclusion

In this study, the differences between the early induction of growth cessation and the depth of endodormancy between two tested poplar cultivars under short photoperiod and low night temperature treatment are associated with the differential expression levels of *CBF1* and *PtDAM1* genes in buds as well as degradation of growth-promoting phytohormones auxin and cytokinins in leaves. However, since other *DAM* genes were not examined, we cannot rule out the possibility of other *DAM* gene involvement.

## Methods

### Plant material and dormancy induction conditions

Two hybrid poplar clones, Walker (*Populus deltoides var. occidentalis* × *Populus petrowskyana*) and Okanese (*P.* ‘Walker’ × *P. petrowskyana*) (kindly supplied by Raju Soolanayakanahally, Agriculture and Agri-Food Canada) were used in this study - under the same growing conditions as described in Kalcsits et al. (2009) [13]. Briefly, hardwood cuttings were planted in KEKKILÄ DSM 3 W (Kekkilä Oy, Finland) propagation media (rich in peat and perlite). Approximately 20 cm long cuttings were placed in each pot (20 × 20 × 20 cm). Before planting, the basal cuttings were dipped in INCIT-8 (Bioplant) rooting stimulating powder. The medium was kept moist during rooting. The hardwood cuttings were grown under LD (18/6 h light/dark) at 22/20 °C and at 75% RH in PGR15 growth chambers (Conviron PGR15, Controlled Environments Ltd., Winnipeg, MB, Canada). Then plants were moved and grown in a greenhouse under natural light at 20 ± 5 °C, fertilized with Peters Professional 20–10-20 (N-P-K) fertilizer (diluted to 100 ppm N) once a week. Only the 4 strongest, healthiest branches were left on each plant. The rooting and growth period lasted two months. Subsequently, when the branches reached 30–40 cm length, the plants were transferred into dormancy inducing conditions in growth chambers of SD conditions (12/12 h, light/dark period) at 18/3 °C temperature for 30 days. The length of the light period was then decreased further to 10-h day length for an additional 30 days (60 days in total). These temperature and light conditions ensured dormancy separation responses between Okanese and Walker [13].

### Dormancy assessment

Dormancy development was measured using the bud-break method adapted from Kalcsits et al. (2009) [13]. In brief, small cuttings with two buds were collected from two pots from each genotype. For each genotype and every sampling time-point, 20 branches were cut, so the budburst on 40 buds was examined at given time-point. Cuttings were put in water in glass tubes and kept under LD conditions (18-h daylength) at continuous 22 °C. Samples were collected in every 10th day over the 60 days long experimental induction period. Bud-break was defined as the point when the first leaves started to emerge from the dormant bud, a longer time to bud-break indicates a higher level (i.e. deeper) dormancy. The depth of dormancy (ΔDBB) was calculated according to Kalcsits et al. (2009) as the difference between the days to bud break between the last and the first sampling days [13].

### Growth cessation assessment

The length of the growing branches was measured from the base to the apex every week. Seven pots with 4 branches were measured per genotype. Growth rates (cm*week^− 1^) were calculated, and when the growth rates (almost) reached zero, the plants were considered to have stopped their growth period. For these examinations, we use different plants than for gene expression and hormone analysis. These plants were not wounded during the whole experiment.

### Gene expression studies

The youngest fully expanded leaf and mid branch bud samples (about 3 plants per every sampling point altogether 12 leaves and buds were collected) for gene expression studies were collected 4–6 h after the start of the photoperiod and frozen immediately in liquid nitrogen and kept at − 80 °C till RNA extraction. Samples were homogenized by TissueLyser II (Qiagen) equipment (29 Hz, 1:30 min), twice. Then 700 μl pre-warmed elution buffer (3% CTAB, 1.4 M NaCl, 200 mM EDTA, 100 mM Tris-HCl, 2% PVPP, 2% β-mercaptoethanol and 80 μg/ml proteinase K) were added to the homogenates. The tubes were kept at 65 °C for 10 min. Then 700 μl phenol-chloroform-isoamyl alcohol (25:24:1) were added. After 5-min centrifugation at 12000 RPM the upper phase was transferred into new tubes. Chloroform-isoamyl alcohol (24:1) was added, and after a new centrifugation step (5 min at 12000 RPM), the RNA was precipitated by the addition of 0.1 volume of Na-acetate (3 M, pH 5.2) and 2 volume of absolute ethyl alcohol. The mixture was uploaded to Direct-zol™ RNA MiniPrep kit columns (Zymo Research, Corp., Irvine, CA, USA) and the RNA isolation process was finished according to the manufacturer’s instructions. The residual DNA was digested by DNase enzyme and pure RNAs were used for cDNA synthesis. cDNAs were transcribed by M-MLV-RT enzyme (Promega Corporation, Madison, WI, USA) and Oligo (dT)_18_ Primers (Thermo Fisher Scientific Inc., Wilmington, MA, USA). 1500 ng RNA were transcribed into cDNA in 25 μl final volume, then diluted to the final volume of 100 μl. 1.0 μl cDNA solution was used for every qRT-PCR. The gene expression levels were determined with the CFX96 Touch™ Real-Time PCR Detection System (Bio-Rad Hungary Ltd., Budapest, Hungary) using the 2x qPCRBIO SyGreen Blue, Mix Separate ROX (PCR Biosystems Ltd., London, United Kingdom) in 10 μl final volume. All the primer sequences, (listed in the Supplemented Table 1), with exception of *PtDAM1,* were collected from the work of Menon et al. (2015) [58]. The normalized relative gene expression levels were calculated by the ΔΔCt method [90]. Ct values were normalized to the Ct values of the housekeeping *Pt18S* rRNA gene (Supp. Table 1). Expression level, measured at a given time point, was compared to the expression level measured on the first day for each genotype. The raw ΔΔCt values are included in the Supplemented Table 2.

The relative expression values (fold change) were converted to log_2_ values, clustered and visualized with the Gitools software on the Supplemented Fig. 2 [91].

### Identification of PtDAM1 gene

For sequence analysis, the *Populus trichocarpa* reference genome assembly was retrieved from the NCBI Assembly server (https://www.ncbi.nlm.nih.gov/assembly) at proteome level (GCF_000002775.4). Pfam and Hidden Markov Model (HMM) based protein domain search was performed using *hmmscan* packages of HMMER 3.0 software [92]. The protein collection from the poplar proteome was aligned using a MUSCLE alignment method (Fig. 5) and inferred using Maximum-likelihood phylogenetic tree by MEGA6 software package [93]. Based on the Bayesian Information Criterion (BIC) the best-fit, Jones-Taylor-Thornton (JTT + G) substitution pattern was chosen for the phylogenetic reconstruction. One thousand bootstrap pseudo-replicates were used to test the reliability of the inferred tree.

### Hormone analysis

The youngest fully expanded leaf samples (ca 50 mg FW) were purified and analyzed according to Dobrev and Kamínek (2002), Dobrev and Vankova (2012) and Svačinova et al. [94–96]. Frozen samples were homogenized and extracted with cold (− 20 °C) methanol/water/formic acid (15/4/1, v/v/v). The following isotope-labelled internal standards (10 pmol/sample) were added: ^13^C_6_-IAA (Cambridge Isotope Laboratories); ^2^H_4_-SA (Sigma-Aldrich); ^2^H_3_-PA, ^2^H_3_-DPA (NRC-PTI); ^2^H_6_-ABA, ^2^H_5_-JA, ^2^H_5_-transZ, ^2^H_5_-transZR, ^2^H_5_-transZ7G, ^2^H_5_-transZ9G, ^2^H_5_-transZOG, ^2^H_5_-transZROG, ^2^H_5_-transZRMP, ^2^H_3_-DZ, ^2^H_3_-DZR, ^2^H_3_-DZ9G, ^2^H_6_-iP, ^2^H_6_-iPR, ^2^H_6_-iP7G, ^2^H_6_-iP9G, ^2^H_6_-iPRMP (Olchemim). Phytohormones were separated with a reverse phase-cation exchange SPE column (Oasis-MCX, Waters) into the acid fraction by elution with methanol [auxins, abscisic acid (ABA), salicylic acid (SA), jasmonic acid (JA)], and into the basic fraction by elution with 0.35 M NH_4_OH in 60% methanol [cytokinins (CKs)]. Fractions were analyzed using HPLC (Ultimate 3000, Dionex) coupled to a 3200 Q TRAP hybrid triple quadrupole/linear ion trap mass spectrometer (Applied Biosystems). Hormone quantification was performed by the isotope dilution method with multilevel calibration curves (r^2^ > 0.99). Data processing was performed with the Analyst 1.5 software package (Applied Biosystems). Raw data are included in the Supplemented Table 3.

### Statistical analysis

One-way ANOVA and Scheffe post hoc test were performed using SPSS 22.0. Because of the unequal variances, Levene’s test, the Brown-Forsythe follow up robust tests of equality of means were used. The principal component analysis (PCA) results were obtained by using the Scikit-learn Python module (version 0.23) [97] and Python 3.8. Following mean and variance standardization of the dataset, linear dimensionality reduction was performed by applying singular value decomposition with six extracted components.

## Supplementary Information

**Additional file 1: Supplemented Figure 1** Relative expression levels in buds (panel A-E) and in leaves (panel F-K) of the *PtCBF1, PtCBF2, PtCBF3, PtCBF4, PtCBF5* and *PtCBF6* genes in the Okanese (black bars) and Walker (white bars) genotypes. The expression levels were determined by the ΔΔCt method. *Pt18S* rRNA gene was used as a housekeeping gene for normalization. Mean expression values were normalized per the expression level at the zero sampling time-point, separately for each genotype. The expression values are presented in log_2_ scale. Error bars represent the ±SEM originating from 3 biological and 3 technical replicates. ‘nd’: the expression level was undetectable.

**Additional file 2: Supplemented Figure 2** Heatmap of the combined phytohormone concentration in leaves (L) and expression of *CBF*s and *DAM* genes (leaves (L) and buds (B)) in ‘Okanese’ (Ok) and ‘Walker’ (Wa) poplar hybrid cultivars over the 60-day short photoperiod and low night temperature growth cessation/dormancy induction treatment. The heatmap was clustered by Euclidean distance. The color bars and letters represent the hierarchy between the investigate gene expression and hormone compound levels.

**Additional file 3: Supplemented Table 1** Sequences, melting temperatures and GC contents of the primers used in the study. All the primer sequences listed, with exception of *PtDAM1,* were collected from the work of Menon et al. (2015) [58].

**Additional file 4: Supplemented Table 2** Raw relative gene expression data of the *CBF* genes.

**Additional file 5: Supplemented Table 3** Raw data of hormone analysis.

## Data Availability

All the relevant data are included in the manuscript and the supplemented materials.

## Abbreviations

ABA: Abscisic acid
ACC: 1-aminocyclopropane-1-carboxylic acid
BA: Benzoic acid
CBF: C-repeat binding factor protein
cZ: cis-zeatin
cZ7G: Cis-zeatin-7-glucoside
cZOG: Cis-zeatin-O-glucoside
cZR: Cis-zeatin riboside
cZROG: Cis-zeatin riboside-O-glucoside
DAM: Dormancy-associated MADS-box protein
DBB: Days to bud break
IAA: Indole-3-acetic acid
iP: Isopentenyl adenine
iP7G: Isopentenyl adenine-7-glucoside
iPRPM: Isopentenyl adenosine monophosphate
JA: Jasmonic acid
Ox-IAA: oxo-IAA
PA: Phaseic acid
PCA: Principal component analysis
SA: Salicylic acid
tZ: Trans-zeatin
tZR: Trans-zeatin riboside
