# Supplementary figures and images for: Elucidation of molecular and hormonal background of early growth cessation and endodormancy induction in two contrasting Populus hybrid cultivars
Source: BMC Plant Biol. 2021 Feb 24;21:111. doi: 10.1186/s12870-021-02828-7 (PMC7905644; doi:10.1186/s12870-021-02828-7)

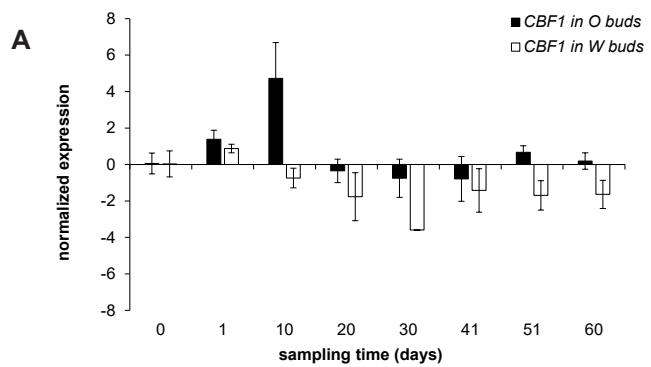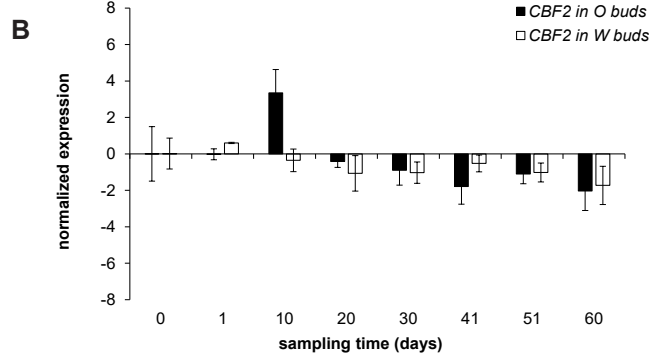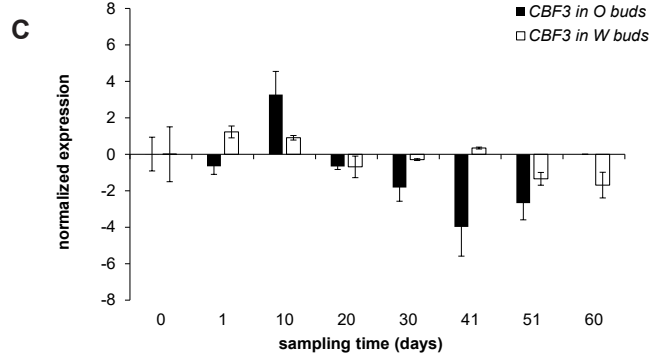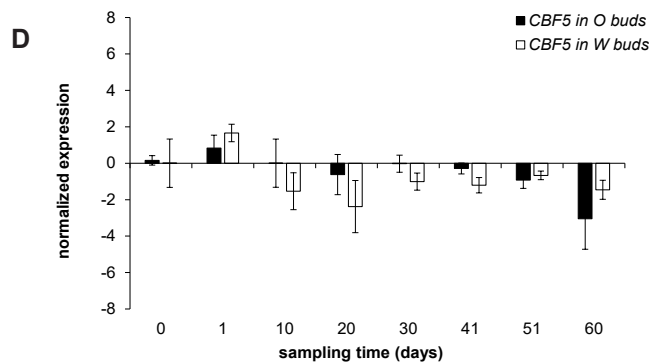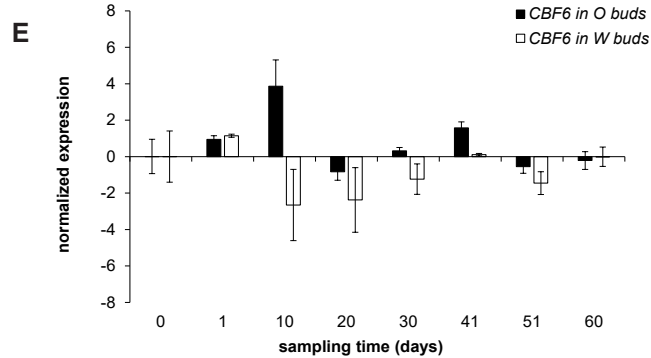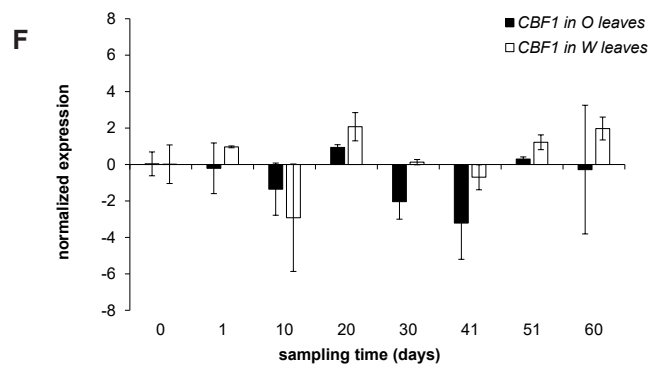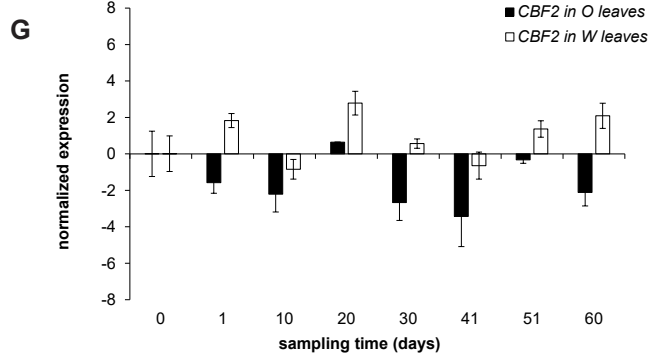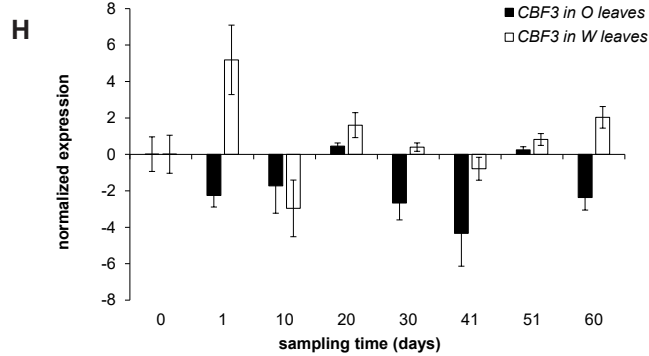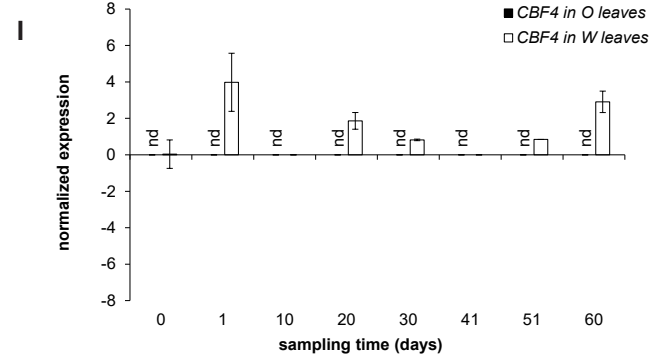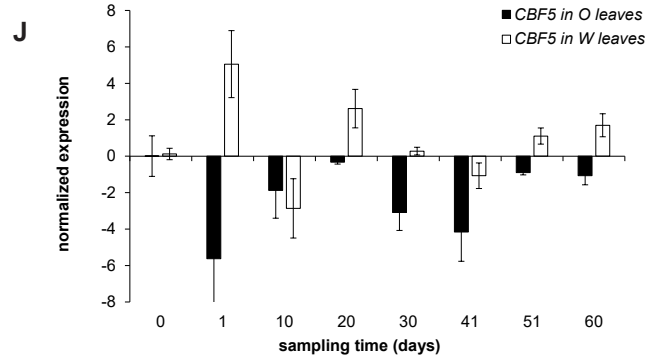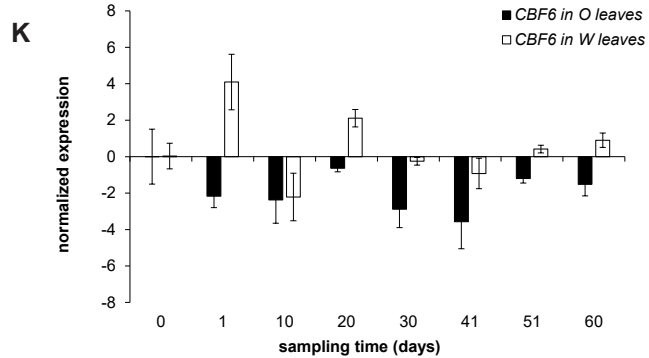

Supplement: Supplementary file 1 — Additional file 1: Supplemented Figure 1 Relative expression levels in buds (panel A-E) and in leaves (panel F-K) of the PtCBF1, PtCBF2, PtCBF3, PtCBF4, PtCBF5 and PtCBF6 genes in the Okanese (black bars) and Walker (white bars) genotypes. The expression levels were determined by the ΔΔCt method. Pt18S rRNA gene was used as a housekeeping gene for normalization. Mean expression values were normalized per the expression level at the zero sampling time-point, separately for each genotype. The expression values are presented in log2 scale. Error bars represent the ±SEM originating from 3 biological and 3 technical replicates. ‘nd’: the expression level was undetectable. [file 12870_2021_2828_MOESM1_ESM.pdf]

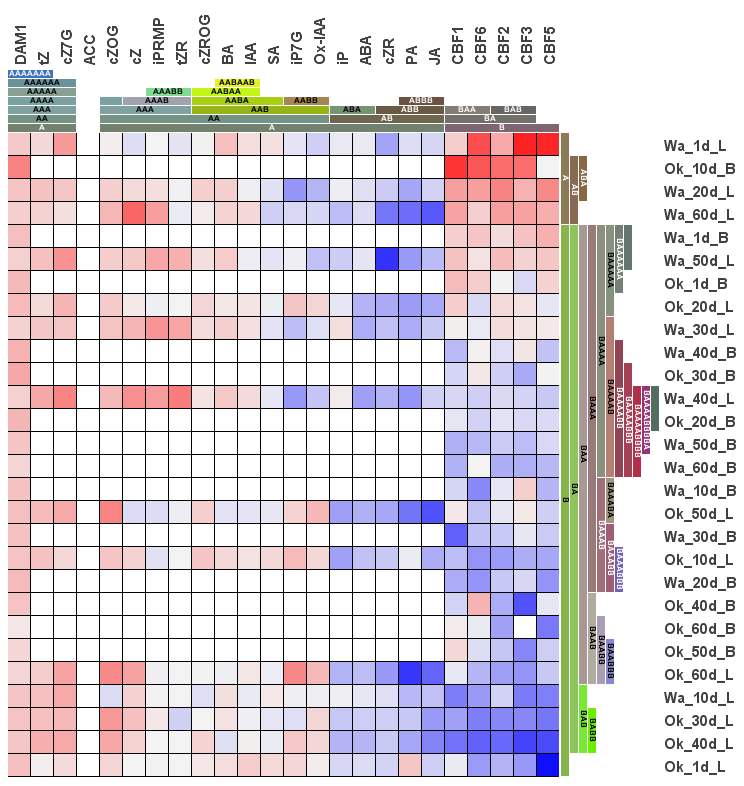

Supplement: Supplementary file 2 — Additional file 2: Supplemented Figure 2 Heatmap of the combined phytohormone concentration in leaves (L) and expression of CBFs and DAM genes (leaves (L) and buds (B)) in ‘Okanese’ (Ok) and ‘Walker’ (Wa) poplar hybrid cultivars over the 60-day short photoperiod and low night temperature growth cessation/dormancy induction treatment. The heatmap was clustered by Euclidean distance. The color bars and letters represent the hierarchy between the investigate gene expression and hormone compound levels. [file 12870_2021_2828_MOESM2_ESM.png]
