# Supplementary material for: Elucidation of molecular and hormonal background of early growth cessation and endodormancy induction in two contrasting Populus hybrid cultivars
Source: BMC Plant Biol. 2021 Feb 24;21:111. doi: 10.1186/s12870-021-02828-7 (PMC7905644; doi:10.1186/s12870-021-02828-7)
Supplement: Supplementary file 3 — Additional file 3: Supplemented Table 1 Sequences, melting temperatures and GC contents of the primers used in the study. All the primer sequences listed, with exception of PtDAM1, were collected from the work of Menon et al. (2015) [58]. [file 12870_2021_2828_MOESM3_ESM.pdf]

Table S1

Sequences, melting temperatures and GC contents of the primers used in the study.

**Primer name**	**Sequence of**					**Amplicon**	
	Forward	Reverse	Length (bp)	Tm (°C)	GC%	Length (bp)	Melt temp. (°C)
PbCBF1	GGAGTTCAACCAGGTGCAAT	ACACAGGATGCCTTGTTTCC	20/20	63.9/63.9	50/50	109	78.0
PbCBF2	GGGAGGTGAGTTGATGAGGA	TATTAGCCAACAACCCTGGC	20/20	64.2/63.6	55/50	128	82.5
PbCBF3	TTTCAAATGAGGCCAAGGAC	CCTCCCTCCTGAAATCTTCC	20/20	63.9/63.8	45/55	105	80.0
PbCBF4	AGGCAAATCTGCTTGCCTTA	ATCATTTTCTCCTGGCATGG	20/20	63.7/63.7	45/45	271	82.0
PbCBF5	CTGAAGGGAACTGCTACTT	GAACAGTGCATTGGGTTGATG	19/21	56.0/65.3	47.3/47.6	396	81.5
PbCBF6	GACCTTCAAGAGCCCAGAAA	CCTTCTCTGAGCAGCAGTTATTA	20/23	63.3/61.5	50/43.4	425	82.0
PtDAM1	CGCCGTCTCTTGAGTTGCAG	GGCCAACCCTAATTTGAGAGAGG	20/23	68.4/67.6	60/51.1	432	84.0
18S	AATTGTTGGTCTTCAACGAGGAA	AAAGGGCAGGGACGTAGTCAA	23/21	65.5/66.7	39.1/52.3	74	82.5
